# L‐Shaped Three‐Portal Configuration for Arthroscopic Double‐Row Suture Repair of Lafosse Type III and IV Subscapularis Tears

**DOI:** 10.1002/atn2.70173

**Published:** 2026-07-14

**Authors:** Gang Li, Feifei Shen, Wei Sun, Shaobo Li, Dedong Cui, Hui Luan, Xingtao Ge

**Affiliations:** ^1^ Department of Orthopedics Qilu Hospital Cheeloo College of Medicine Shandong University Qingdao China; ^2^ Department of Pathology Qilu Hospital Cheeloo College of Medicine Shandong University Qingdao China

## Abstract

Lafosse type III and IV subscapularis tendon injuries are severe rotator cuff tears. Current repair uses anterolateral, anterior, and lateral portals, which have restricted visualization and difficult maneuverability. This technical note describes an L‐shaped three‐portal (LST) combination: anterosuperolateral, anteroinferolateral, and anteroinferomedial portals. The L‐shaped three‐portal configuration establishes an unobstructed surgical field free from bony interference, allowing comprehensive subscapularis tendon exposure. It optimizes anchor insertion angles and suture passing path, promoting accurate anchor fixation and effective suture management. Ultimately, it minimizes surgical complications and improves double‐row repair efficiency for Lafosse type III/IV injuries, presenting a secure and efficient approach. This article outlines the technique, highlighting procedural aspects, potential risks, advantages, and limitations.

## Graphical abstract

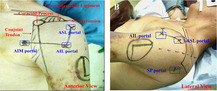

**VIDEO 1:** The authors' information, disclosure, surgical approach establishment and surgical technique. Operative side: left shoulder; Position: right lateral decubitus. SP portal: standard posterior portal. ASL portal: Anterosuperolateral portal. AIL portal: Anteroinferolateral portal. AIM portal: Anteroinferomedial portal. AP: Arthroscopic portal, red arrow. OP: Operative portal, green arrow. Video content can be viewed at https://doi.org/10.1002/atn2.70173.

Subscapularis tendon injury, a significant rotator cuff pathology, causes anterior shoulder pain, marked reduction in active internal rotation strength, and functional limitation. The Lafosse classification provides a clear framework for diagnosing injury severity and guides treatment decisions in clinical practice.^1^, ^2^, ^3^ Although current research findings cannot yet definitively determine which repair construct (single‐row or double‐row) is superior for subscapularis tendon tear repair,^4^ it is currently widely believed that Lafosse type III and IV subscapularis tendon injuries require arthroscopic double‐row suture fixation to restore tendon integrity and function.^2^, ^5^, ^6^, ^7^ Double‐row fixation disperses stress applied to the suture points, reduces rerupture risk, and enhances the stability and tensile strength of the suture.^6^, ^8^ However, traditional portals limit visualization of the subscapularis tendon footprint, making double‐row fixation difficult. Due to limited visualization, most orthopaedic surgeons reluctantly opt for single‐row fixation, and some surgeons even cut the long head of the biceps tendon to improve visualization.

Studies have shown that the choice of surgical approach significantly influences the surgical outcome, postoperative recovery, and the incidence of complications.^9^, ^10^ Therefore, selecting an appropriate combination of arthroscopic portals is crucial for ensuring the safety of subscapularis tendon repair, enhancing repair quality, and increasing the success rate.^11^ Currently, the classic arthroscopic portal combinations used to repair Lafosse type III and IV subscapularis tendon injuries include anterolateral, anterior, and lateral portals.^12^, ^13^, ^14^ However, these combinations often provide inadequate exposure and limited operative space, causing imprecise anchor positioning and suturing. This compromises repair accuracy, raises iatrogenic injury risk, and may result in incomplete repair or omission.^11^

The arthroscopic view is often obstructed by three anatomical structures: the humeral head, coracoid process, and conjoint tendon. The L‐shaped three‐portal (LST) layout—anterosuperolateral, anteroinferolateral, and anteroinferomedial portals—overcomes these limitations by avoiding anatomical obstruction and providing a clearer and more stable view. This facilitates precise anchor implantation and suture threading for double‐row subscapularis repair, reducing the risk of neurovascular injury, and improving biomechanical strength.^15^, ^16^ This article details the technical design and operative steps of this portal layout for clinical reference and application.

## SURGICAL TECHNIQUES

### Preparation

Patients with Lafosse type III and IV subscapularis tendon injuries underwent arthroscopic repair under general anesthesia in lateral decubitus position (shoulder flexed 20°, abducted 40°) using a 30° arthroscope. Table 1 summarizes surgical techniques and considerations; Table 2 outlines advantages and disadvantages. Ethics committee approval and informed consent were obtained.

**TABLE 1:** Surgical Pearls and Pitfalls of the LST Portal Combination

Pearls
(1) Using a 30° endoscope via the ASL approach allows for comprehensive visualization, clearly visualizing anatomical features such as the lesser tuberosity of the humerus and the axillary nerve. (2) A high‐strength suture is introduced into the ruptured end of the subscapularis tendon to facilitate traction of the tendon, which contribute to expand the visual field. (3) Before the establishment of the AIM and AIL portals, it is essential to use a spinal needle to perform initial localization and enhance the accuracy of approach establishment.

Pitfalls
(1) Too lateral placement of the surgical drapes can affect the establishment of the AIL approach. The edge of the drape should be located between the midclavicular line and the sternal midline. (2) Prolonged surgical time can lead to extravasation of irrigation fluid into the pectoralis major space, causing severe swelling and potentially resulting in fluid overload. Therefore, the surgical time should be kept within 90 minutes if possible. (3) When establishing the AIM approach, it is essential to fully expose the anterior interval of the subscapularis tendon. Blind insertion of the spinal needle without adequately dissecting the interval can lead to a pneumothorax.

AIL, anteroinferolateral; AIM, anteroinferomedial; ASL, anterosuperolateral; LST, L‐shaped three‐portal.

**TABLE 2:** Advantages and Disadvantages of the LST Portal Combination

Advantages
(1) The LST portal configuration provides multiview visualization, which allows the surgeon to clearly identify the extent and depth of the subscapularis tendon tear. (2) This configuration offers a large operating space that allows flexible movement of instruments, enabling accurate suturing, and minimizing interference with surrounding soft tissues. (3) It reduces the risk of injury to surrounding structures, such as the long head of the biceps tendon and other parts of the rotator cuff, thereby promoting early postoperative recovery.

Disadvantages
(1) This procedure demands a high level of surgical skill. The surgeon must be proficient in local anatomy and possess extensive arthroscopic experience; otherwise, the surgical time may increase, or procedural errors may occur. (2) Although designed to minimize trauma, the establishment of multiple surgical approaches may still pose a potential risk of neurovascular injury.

LST, L‐shaped three‐portal.

### Surgical Approach Establishment

**FIGURE 1:**
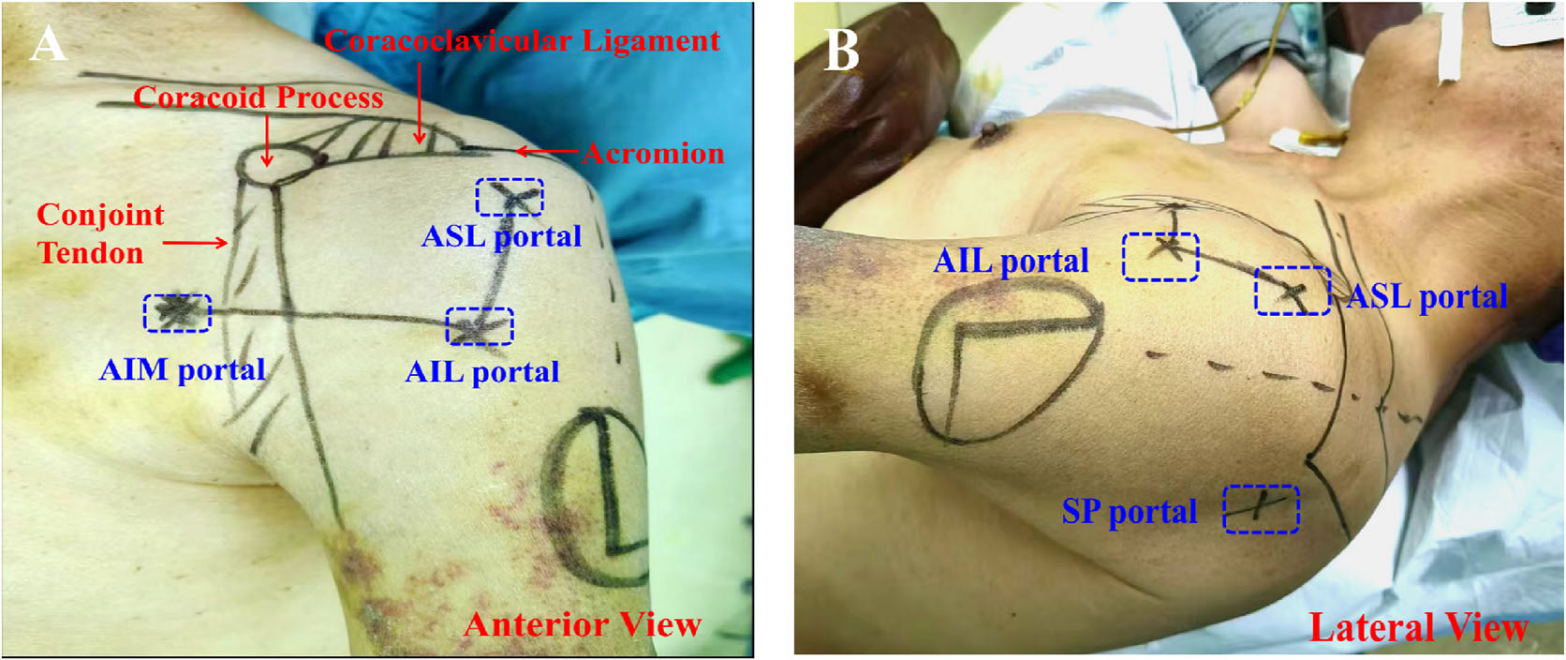
The LST portal configuration (A: anterior view, B: lateral view). The patient was diagnosed with a left rotator cuff tear and positioned in the lateral decubitus position during the procedure. Operative side: left shoulder; Position: right lateral decubitus. (AIL portal, anteroinferolateral portal; AIM portal, anteroinferomedial portal; ASL portal, anterosuperolateral portal; LST, L‐shaped three‐portal; SP portal, standard posterior portal.)

Standard posterior portal: Positioned 2 cm distal and 1 to 2 cm medial to the posterolateral acromion corner for the overall joint visualization and subscapularis tendon injury classification. The LST portal layout (for Lafosse type III and IV repairs) combines three portals: anterosuperolateral (ASL), anteroinferolateral (AIL), and anteroinferomedial (AIM) portals (Figure 1A,B). The ASL portal is located anterior to the biceps tendon and lateral to the anterolateral acromial edge and serves as a subacromial observation portal for the subscapularis tendon and for passing high‐strength sutures to maintain tension on the subscapularis tendon stump. The AIL portal is positioned approximately 3 fingerbreadths transversely below the ASL portal, parallel to the subscapularis tendon upper edge and enables 270° release of the subscapularis tendon stump and adhesions, tendon suturing, and knot tying. The AIM portal is situated about 2 cm medial to the conjoint tendon and parallel to the AIL portal and is used for anchor implantation and suturing of the subscapularis tendon with a lasso suture device.

### Surgical Technique

**FIGURE 2:**
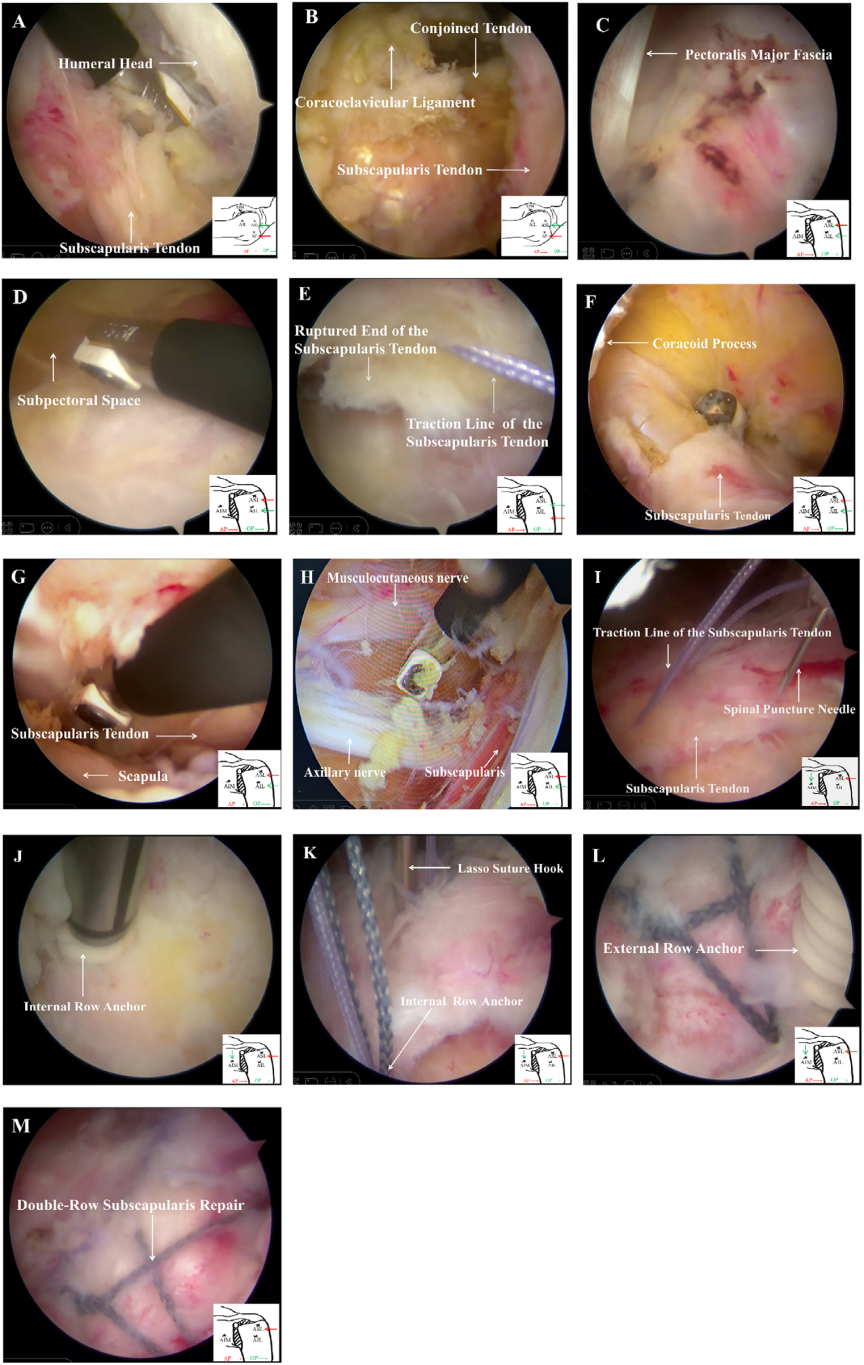
Double‐row suture repair of type III and IV subscapularis tendon injuries under the LST approach. (A,B) Expose the subscapularis tendon (A), coracohumeral ligament, and conjoint tendon (B). (C,D) Expose the interval between coracoid, conjoined tendon, coracoclavicular ligament, and pectoralis major. (E) Subscapularis tendon traction with reinforcement suture. (F‐H) A 270° subscapularis tendon adhesiolysis. (I) Establishment of the AIM portal. (J) Insertion of the inner‐row anchor. (K) Horizontal mattress suture of the subscapularis tendon. (L) Insertion of the outer‐row anchor. (M) Double‐row suture of the subscapularis tendon. Operative side: left shoulder; Position: right lateral decubitus. (AIL portal, anteroinferolateral portal; AIM portal, anteroinferomedial portal; AP, arthroscopic portal, red arrow; ASL portal, anterosuperolateral portal; LST, L‐shaped three‐portal; OP, operative portal, green arrow; SP portal, standard posterior portal.)

First, establish the standard posterior portal to visualize the joint with a 30° arthroscope. Then, create the ASL portal to perform rotator interval debridement using a shaver and a plasma ablation device. This exposes the subscapularis tendon (Figure 2A), coracohumeral ligament, and conjoint tendon (Figure 2B). Next, assess the subscapularis tendon tear, identify the “comma sign,” a full‐thickness subscapularis tendon tear indicator, and repair or debride associated cartilage, labrum, and biceps injuries.

Insert the arthroscope through the ASL portal for observation and then establish the AIL portal via spinal needle for bursectomy. The coracoid process is identified by using the coracohumeral ligament as a landmark. Locate the interval between the coracoid, conjoined tendon, coracoclavicular ligament, and pectoralis major; incise the pectoralis major fascia (Figure 2C). Expand the space proximally to the mid‐coracohumeral ligament, distally to the superior pectoralis major humeral insertion, and medially to the coracoid 's inner edge and pectoralis minor surface (Figure 2D). This creates a spacious working area which is conducive to subsequent operations. Expose the subscapularis tendon footprint on the humerus, avoiding injury to the transverse humeral ligament if the biceps tendon is intact. Use a Lasso suture passer to place a reinforcement suture at the subscapularis stump for traction (Figure 2E), and pull the suture through the ASL portal to maintain tension. For severely retracted tendons, perform a 270° release of subscapularis adhesions superiorly, anteriorly, and posteriorly to facilitate mobilization. Debride the coracoid to its medial edge (Figure 2F). Release adhesions between the anterior scapula and subscapularis muscle (Figure 2G), and between the subscapularis tendon and conjoined tendon, carefully protecting the axillary and musculocutaneous nerves (Figure 2H, a Lafosse type IV case).

Then, the AIM portal is established with spinal needle guidance (Figure 2I). Through this portal, surgeons place inner and outer row anchors perpendicular to the lesser tubercle footprint and suture the subscapularis tendon, effectively avoiding coracoid interference. After lesser tubercle preparation, implant the lowest inner row anchor first at 45° to cortical bone, 2 mm from cartilage (Figure 2J). The anchors are spaced 1 cm apart. Place a horizontal mattress suture 2 to 3 mm from the tendon tear edge and secure it (Figure 2K). Insert the outer row anchor 1 cm lateral to the inner row to fix the suture line (Figure 2L), adjusting optimal positions through internal and external rotation of the upper arm. After double‐row subscapularis repair, reinsert the arthroscope into the subacromial space (Figure 2M) and glenohumeral joint to verify ideal anatomical reattachment. The specific surgical technique is shown in Video 1.

## DISCUSSION

Lafosse type III and IV subscapularis tendon injuries are considered severe types of rotator cuff injuries, often accompanied by significant functional loss and biomechanical imbalance, and they represent a challenge in repair within the fields of sports medicine and arthroscopic surgery.^2^, ^5^ Arthroscopic repair is now the most common method, offering minimally invasive precise visualization and ability to address associated lesions like biceps tendon injuries. Lanz et al.^5^ showed that arthroscopic repair of type III and IV tears significantly improves clinical scores, maintains muscle strength, ensures tendon structural integrity, and reduces retear rates to only 11%.

For Lafosse type III and IV subscapularis tendon injuries, multiple portals are essential^17^ but existing methods face visualization limitations.^11^, ^12^ The most common repair approach combines standard lateral, anterior, and anterolateral portals^12^, ^13^ yet suffers from high technical difficulty, limited visualization, and difficult instrumentation, often resulting in incomplete repair or omitted tendon tissue.^11^ Consequently, most surgeons are forced to adopt suboptimal single‐row suture repair. This article describes a LST configuration specifically designed for double‐row suture repair of severe injuries. The LST layout provides unobstructed operative space through strategically positioned portals, fundamentally optimizing the surgical view. This arrangement markedly enhances subscapularis tendon exposure and instrumentation efficiency, reduces surrounding soft tissue damage, and makes the originally difficult and complex double‐row suture repair relatively simpler.

The LST portal configuration offers several key advantages. First, it provides multiview visualization capabilities, enabling surgeons to clearly identify the extent and depth of subscapularis tendon tears; this clear identification facilitates the perpendicular implantation of anchors placed in the medial and lateral rows on the lesser tuberosity footprint. Second, the LST portal design creates a large operating space for flexible instrument movement, precise suturing, and efficient suture management, while reducing soft tissue interference. Finally, it minimizes injury risk to adjacent structures like the long head of the biceps brachii tendon and other rotator cuff components, thus promoting early functional recovery. However, disadvantages exist. The technique demands high surgeon proficiency in regional anatomy and extensive arthroscopic experience; otherwise, surgical time may increase, and errors may occur. Although designed to minimize trauma, establishing multiple portals still carries potential neurovascular injury risks, including inadvertent damage to the axillary nerve, musculocutaneous nerve, and adjacent vessels.

The LST approach enables minimally invasive double‐row repair of Lafosse III and IV subscapularis tears, improving surgical exposure, operative efficiency, and reducing complications. Although future large‐scale clinical trials are needed to establish long‐term efficacy evidence, this technique provides a viable option that advances subscapularis repair, requiring individualized evaluation of patient anatomy and surgeon skill.

## DISCLOSURES

The authors (G.L., F.S., W.S., S.L., D.C., H.L., X.G.) declare that they have no known competing financial interests or personal relationships that could have appeared to influence the work reported in this article.
